# Healthcare barriers, what about older age? A comment on Malik-Soni et al.

**DOI:** 10.1038/s41390-021-01596-2

**Published:** 2021-06-09

**Authors:** David Mason

**Affiliations:** Social, Genetic, and Developmental Psychiatry Centre, Institute of Psychiatry, Psychology, and Neuroscience, King’s College London, London, UK

Autism is a neurodevelopmental condition; characteristics of autism comprise difficulties with social communication (e.g., atypical social gaze or poorly integrated verbal and nonverbal communication^1^), and the presence of restricted interests and/or repetitive behaviours (e.g., repetitive lining up of objects, insistence on routines, and fixated interests of acute intensity^1^).^2^ A number of recent quantitative studies have found that autistic adults are at risk of poorer health outcomes. [A recent study by Kenny et al.^3^ reported that there was no consensus on how to refer to those with an autism diagnosis. Hence, “autistic individual(s)”, “autistic person”, or “people on the autism spectrum” will be used interchangeably throughout.] First, the rates of a range of physical health conditions are elevated among samples of autistic adults compared to matched comparison samples. A study comparing health insurance records for patients in America (*N* = 1507 people on the autism spectrum and 15,070 non-autistic people matched on age and sex) found markedly elevated rates of most conditions, including anxiety (odds ratio, OR = 3.7), dementia (OR = 4.4), cardio-vascular disease (OR = 2.5), neurological diseases (OR = 2.2), sleep disorders (OR = 1.9), and nutritional conditions (i.e. metabolism or deficiency; OR = 2.7).^4^ A follow-up study, with those aged 65+ (*N* = 4685 autistic individuals and *N* = 46,850 matched participants) found broadly the same pattern of results.^5^ These data are consistent with Scottish census data measuring somewhat different conditions (e.g., global reports of physical disability or mental health conditions); those on the autism spectrum were at greater odds of having these conditions.^6^ Second, autistic adults are more likely to die prematurely. Hirvikoski et al.^7^ compared 27,122 autistic individuals (*N* = 6240 with an intellectual disability and *N* = 20,882 without) to 2,672,185 comparison participants. The autistic group died, on average, 16.3 years younger than the comparison group (53.9 versus 70.2 years). This was more pronounced in the autistic group with intellectual disability (mean age of death, 39.5 years) compared to the autistic group without (mean age of death, 58.4 years). Odds ratios for all ICD-10 codes examined in the study [infections, neoplasms, endocrine, mental and behavioural disorders, nervous system, circulatory system, respiratory system, digestive system, genitourinary system, symptoms/signs/abnormal findings/other, suicide, external causes/other, and other] were higher for the autistic group, except for death by infection (OR = 1.83): for example, conditions of the nervous system (OR = 7.5), respiratory system (OR = 2.7), and suicide (OR = 7.6). In the autism group these odds ratios were further elevated for those with an intellectual disability, and also for females. Clearly, these data indicate that autistic individuals are at risk of severely poor health outcomes.

It is not implausible that an inability to access healthcare effectively is related to the outcomes described above. To date, a number of reviews have examined this issue. Mason and colleagues reported that communication and healthcare professionals were two commonly reported barriers. The former reflected characteristics of the autistic person (e.g. atypical communication style or literal interpretations) and the latter—crucially—reflected the (in)flexibility of healthcare providers (e.g. being open to allowing written communication or using accessible language).^8^ Bradshaw et al.^9^ reported that, among other factors, limited services and referral pathways, accessibility of facilities, socioeconomic factors, and previous experiences with healthcare professionals could negatively impact autistic people’s healthcare access.

The recent review by Malik-Soni et al.^10^ makes several important contributions to the literature on healthcare needs and barriers. The authors identify six broad domains of barriers encountered by people on the autism spectrum: shortages of services, lack of clinician knowledge, cost, family and individual knowledge, language, and stigma. Malik-Soni et al. then go on to identify healthcare needs across the stages of life, namely childhood (0–17 years), transition (16–25 years), and adulthood (18+ years). Identifying that urbanicity is a factor in healthcare is important, with those living in rural areas less likely to access healthcare. The consequences for families living in rural areas can impede access to adequate healthcare, including long waits and service shortage. Crucially, families that do not have adequate access to services are less likely to seek help due to cost, time, or the need to take time off from work. This will likely impact some families disproportionately, as those with a lower socioeconomic status or from ethnic minorities are more likely to lack access to services.^11^ Awareness of autism is another important factor related to accessing healthcare. Malik-Soni and colleagues identify that many healthcare professionals report a lack of training about autism and lack confidence to manage the care of autistic patients. Yet, awareness of autism may also be moderated by parent experience and socioeconomic status (i.e. first-time parents and those from lower socioeconomic families are less likely to spot developmental atypicalities).^10^ Hence, removing this barrier is important, as early interventions for autistic children have shown some promise in improving social and emotional behaviours^12,13^ (yet, more work is needed around specific theories of intervention development and understanding mechanisms to translate interventions into real-world settings^14^). The authors also highlight the scarcity of research into the healthcare needs of autistic adults. Malik-Soni and colleagues suggest that there is an urgent need for more research investigating long-term access to healthcare, the impact of co-occurring conditions, medication, and cognitive decline. Vogan et al.^15^ conducted a longitudinal examination of healthcare access with 40 participants (mean age 35.9 years). The most accessed service over the course of the study was the family doctor (97.5%). The Vogan study found that navigating the health system (e.g. knowing where to find help) was the most common barrier. However, consistent with Malik-Soni, almost half the autistic adults (47.4%) reported negative experiences with professionals; this could be ameliorated with more awareness and training about autism for health professionals (Malik-Soni et al.’s second recommendation^10^).

Malik-Soni and colleagues’ review suggests two important factors that could help raise the health outcomes for autistic people: accessing healthcare online and the consequences of aging. One striking finding is the potential utility of accessing healthcare online. Developing remote healthcare access for autistic people is likely to offer a number of benefits. Obvious benefits include the reduction in travel, waiting in aversive environments, and interaction with staff.^16,17^ Autistic people report “camouflaging” in face-to-face interactions—deliberately hiding their autistic characteristics in order to “play the appropriate role” or to avoid appearing “too autistic”.^18^ Camouflaging has been reported, by autistic people, to be tiring and distressing and camouflaging has also been correlated with greater depressive symptomatology.^18,19^ A second, related construct is compensation, whereby autistic people use conscious, cognitive, and effortful strategies to account for difficulties with social communication.^20^ The use of technology as reported by Malik-Soni and colleagues (e.g. connecting physicians to autism experts) could be adapted to try and mitigate these difficulties. Malik-Soni and colleagues describe how telehealth and videoconferencing have led to improving access to healthcare; these approaches could be further leveraged to make all aspects of healthcare more accessible to autistic people. For example, it is plausible that an autistic person may be less inclined to camouflage or compensate if they are at home and can conduct the telehealth appointment via text communication.

Aging research is also in its infancy for autistic people. Although not explicitly addressed in this review (as adulthood is defined as 18+), it is vital to consider how these findings apply to older autistic people. Many autistic people—independent of IQ status—may be dependent on parents,^21,22^ and with increasing age, older autistic people will have still older parents. With subsequent bereavement many older autistic people may therefore lose a vital support system. Hence, the barriers identified here are likely to be compounded for those who are older and supported by their families. As noted earlier, autistic people often have greater comorbidity and are more likely to die early. Given that age is associated with decreasing physical health, it is vital to ask—are these barriers more pronounced in older age?

While a lot of empirical research has looked at the transition from school age into early adulthood (often finding a severe drop in service provision for those autistic individuals leaving the school system),^11,23^ there is very little research examining the transition into residential care in old age. Crompton et al.^24^ have explored this transition, in collaboration with older autistic people, their immediate family, service providers, clinicians, and researchers in the United Kingdom. Themes consistent with Malik-Soni and colleagues’ review were identified, for example, the importance of autism training for residential care staff and recognising autism-related differences. However, it is crucial to extend the recommendations identified by Malik-Soni and colleagues as, in the general population, residential care can adversely impact healthcare. For example, those in residential care often have complex health needs, and healthcare staff may struggle to meet the needs of these individuals.^25^

Thus, Malik-Soni and colleagues’ review adds to our understanding of the barriers that autistic adults face when accessing healthcare. They draw attention to the importance of raising awareness of autism among healthcare professionals and the wider population. Their review highlights the need for longitudinal research and the putative benefits that technology could have to improve the health status of autistic adults. While covering the full lifespan of autistic people, there are some intriguing areas where future research is sorely needed, most notably regarding autism in old age.
