# Correction: Healthcare barriers, what about older age? A comment on Malik-Soni et al.

**DOI:** 10.1038/s41390-021-01652-x

**Published:** 2021-08-19

**Authors:** David Mason

**Affiliations:** Social, Genetic, and Developmental Psychiatry Centre, Institute of Psychiatry, Psychology, and Neuroscience, King’s College London, London, UK

Correction to: *Pediatric Research* 10.1038/s41390-021-01596-2, published online 09 June 2021

The reference list for this Commentary is incorrect from ref. ^11^ onwards. The in-text citations are not affected. The original article has been corrected. The correct references are:

11. Shattuck, P. T., Wagner, M., Narendorf, S., Sterzing, P. & Hensley, M. Post–high school service use among young adults with an autism spectrum disorder. *Arch. Pediatr. Adolesc. Med.* **165**, 141–146 (2011).

12. Hillman, H. Child-centered play therapy as an intervention for children with autism: a literature review. *Int. J. Play Ther.* **27**, 198 (2018).

13. Fuller, E. A. & Kaiser, A. P. The effects of early intervention on social communication outcomes for children with autism spectrum disorder: a meta-analysis. *J. Autism Dev. Disord.* **50**, 1–18 (2019).

14. Vivanti, G. et al. Implementing and evaluating early intervention for children with autism: Where are the gaps and what should we do? *Autism Res.* **11**, 16–23 (2018).

15. Vogan, V., Lake, J. K., Tint, A., Weiss, J. A. & Lunsky, Y. Tracking health care service use and the experiences of adults with autism spectrum disorder without intellectual disability: a longitudinal study of service rates, barriers and satisfaction. *Disabil. Health J.* **10**, 264–270 (2017).

16. Saqr, Y. et al. Addressing medical needs of adolescents and adults with autism spectrum disorders in a primary care setting. *Autism* **22**, 51–61 (2018).

17. Nicolaidis, C. et al. “Respect the way I need to communicate with you”: healthcare experiences of adults on the autism spectrum. *Autism* **19**, 824–831 (2015).

18. Hull, L. et al. “Putting on my best normal”: social camouflaging in adults with autism spectrum conditions. *J. Autism Dev. Disord.* **47**, 2519–2534 (2017).

19. Lai, M. C. et al. Quantifying and exploring camouflaging in men and women with autism. *Autism* **21**, 690–702 (2017).

20. Livingston, L. A. et al. Good social skills despite poor theory of mind: exploring compensation in autism spectrum disorder. *J. Child Psychol. Psychiatry* **60**, 102–110 (2019).

21. Skinner, C. et al. Autistic disorder: a 20 year chronicle. *J. Autism Dev. Disord.* **51**, 677–684 (2021).

22. Howlin, P., Moss, P., Savage, S. & Rutter, M. Social outcomes in mid-to later adulthood among individuals diagnosed with autism and average nonverbal IQ as children. *J. Am. Acad. Child Adolesc. Psychiatry* **52**, 572–581 (2013).

23. Anderson, K. A., Sosnowy, C., Kuo, A. A. & Shattuck, P. T. Transition of individuals with autism to adulthood: a review of qualitative studies. *Pediatrics* **141**, S318–S327 (2018).

24. Crompton, C. J., Michael, C., Dawson, M. & Fletcher-Watson, S. Residential care for older autistic adults: insights from three multiexpert summits. *Autism Adulthood* **2**, 121–127 (2020).

25. Robbins, I. et al. Explaining the barriers to and tensions in delivering effective healthcare in UK care homes: a qualitative study. *BMJ Open* **3**, e003178 (2013).

**Publisher’s note** Springer Nature remains neutral with regard to jurisdictional claims in published maps and institutional affiliations.

